# Acute Hematogenous Osteomyelitis Resulting in Atraumatic Pediatric Forearm Compartment Syndrome

**DOI:** 10.5435/JAAOSGlobal-D-21-00228

**Published:** 2022-06-02

**Authors:** Nichole M. Shaw, Alexander Kish, Raymond Pensy

**Affiliations:** From the University of Maryland Medical Center (Dr. Shaw, Dr. Kish, and Dr. Pensy) and the R. Adams Cowley Shock Trauma Center, Baltimore, MD (Dr. Shaw, Dr. Kish, and Dr. Pensy).

## Abstract

Acute hematogenous osteomyelitis is well described after minor trauma in the pediatric population, with an incidence of 1 to 13 cases per 100,000 individuals. Compartment syndrome (CS) in children is a rare, but potentially devastating disease, classified as “cannot miss diagnosis.” Compared with adults, CS may exhibit a variable presentation with a wide range of symptoms in children, often leading to delayed diagnosis. Expeditious diagnosis and treatment of CS is paramount in minimizing adverse sequelae and maximizing potential functional outcome, regardless of etiology. Here, we present a rare case of atraumatic CS resulting from ruptured subperiosteal abscess secondary to acute hematogenous osteomyelitis in a pediatric male patient with 2 weeks of forearm pain and evolving neurologic deficits with initial delay in presentation to our facility. The ramifications of delayed diagnosis or misdiagnosis of CS emphasize the importance of a high index of suspicion despite atypical presentations in the pediatric patient.

Pediatric forearm compartment syndrome (CS) is a rare but potentially devastating disease.^1,2,3,4^ Common causes include skeletal trauma, intravenous catheter infiltration, intracompartmental hemorrhage, bleeding disorders, and rarely, infection.^5,6^ Regardless of etiology, increased pressure within the forearm compartments results in progressive muscle and nerve ischemia and eventual necrosis. Resultant Volkmann contracture results in profound loss of function, and in rare cases, loss of limb.^6-8^ Rapid diagnosis and management are crucial in minimizing or preventing potentially devastating complications. Herein, we present, we present an unusual case of atraumatic forearm CS following ruptured subperiosteal abscess resulting from acute hematogenous OM. This case report highlights the broad range of potential etiologies and diverse spectrum of presenting symptoms in pediatric CS.

## Case Description

**Figure 1:**
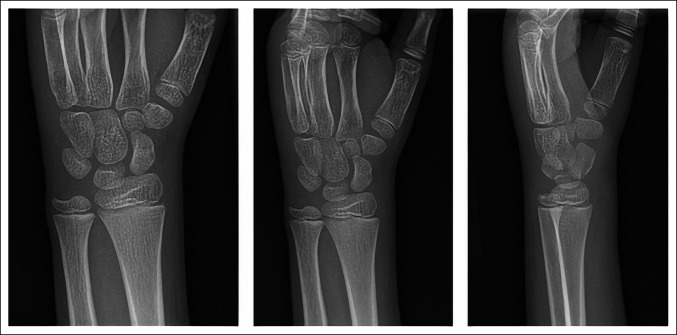
AP, oblique, and lateral radiographs of the left wrist 2 weeks before presentation to our emergency department.

Two and a half weeks before presentation, an otherwise healthy, 10-year-old, African American boy fell forward onto an outstretched left arm while running, experiencing mild wrist pain without deformity. The following day, he presented to his pediatrician with pain and swelling despite cold therapy and anti-inflammatories. Injury radiographs (Figure 1) were normal. He developed a low-grade fever with runny nose 2 days after initial injury with spontaneous resolution. One week later, he experienced atraumatic ipsilateral elbow swelling and pain. He sought follow-up care at an urgent care where he had normal elbow and wrist radiographs. They trialed NSAIDs, cold therapy, and compressive wrapping without improvement over the following week. He ultimately developed an inability to move his elbow, prompting presentation to our emergency department at his pediatrician's recommendation.

**Figure 2:**
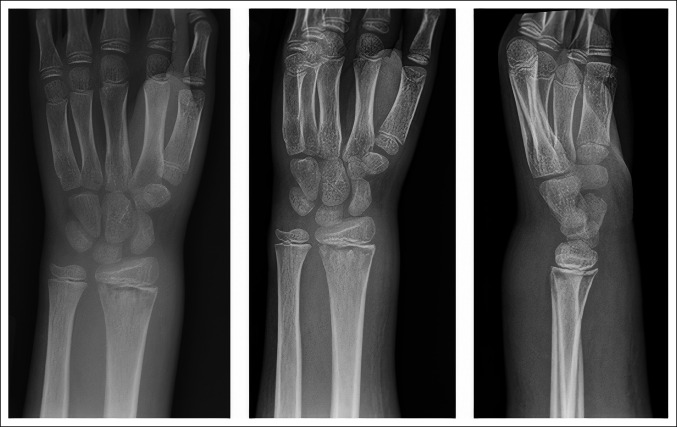
AP, oblique, and lateral radiographs at the time of presentation to our emergency department; diffuse palmar and dorsal soft-tissue swelling noted about the wrist and forearm with mottled appearance of the distal radius physis.

**Figure 3:**
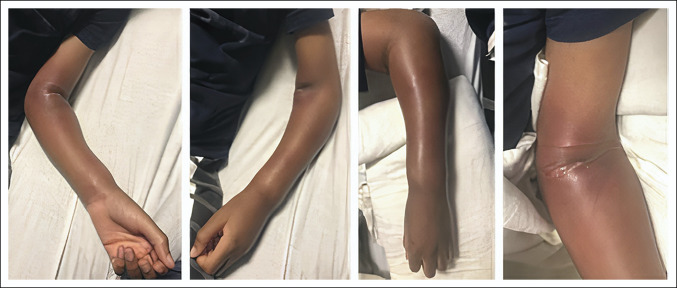
Photographs showing that the elbow and forearm on presentation were red, hot, and diffusely swollen. Anterior, posterior, and mobile wad compartments were tight with palpation.

Radiographs demonstrated subcutaneous swelling and mottling of the distal radius physis, suggesting ongoing infection and prompting orthopaedic consultation (Figure 2). Examination revealed marked swelling circumferentially about the forearm and elbow (Figure 3). The elbow was held flexed at 110° and was unable to range without severe pain passively or actively. The wrist range of motion was limited to 20° of passive flexion and extension because of swelling and pain. He held his fingers in a semiflexed position and had significant forearm pain with passive finger extension. His palmar and dorsal forearm were tense and painful with palpation. His sensation was intact in the radial, median, and ulnar nerve distributions. He had intact ulnar nerve motor function; however, he exhibited posterior interosseous nerve (PIN) weakness and a complete inability to flex the thumb at the interphalangeal joint indicating anterior interosseous nerve (AIN) dysfunction. Laboratory studies were universally elevated: white blood cell count 16.2 k/mcL (normal institutional range: 4.5 to 13.5 k/mcL), erythrocyte sedimentation rate 120 mm/hr (zero to 10 mm/hr), and C-reactive protein (CRP) 15.6 mg/dL (≤1 mg/dL). Compartment pressures were not measured because the clinical diagnosis was clear from his pain with passive finger extension, motor palsy, and tense compartments. CS with evolving neurologic deficits was diagnosed clinically, and he was indicated for emergent forearm fasciotomies.

**Figure 4:**
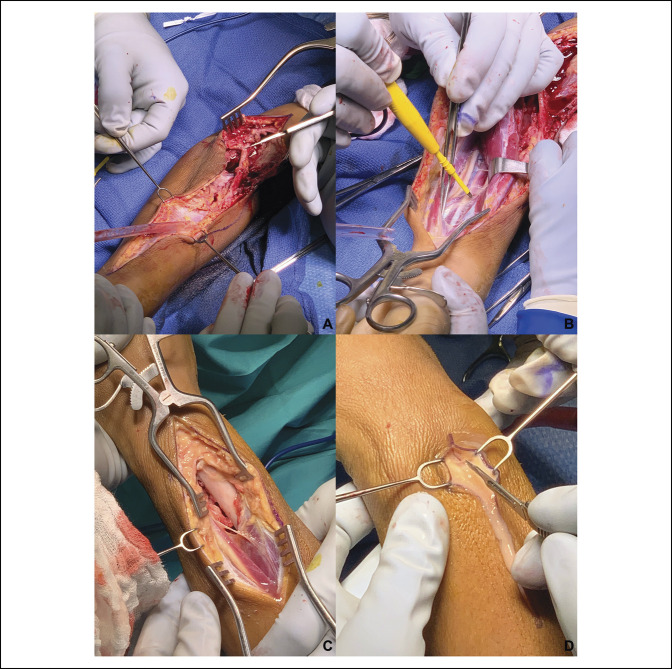
Photographs showing intraoperative findings. **A**, An extensile volar approach was utilized, with abscess noted immediately on incision in antecubital fossa. **B**, The interval between flexor digitorum superficialis and flexor digitorum profundus, exposing the median nerve, just before abscess exposure. **C**, Bare distal one-third radius with denuded periosteum, necrotic pronator quadratus, and disruption of the interosseous membrane. **D**, Dorsal skin incision with frank purulence encountered immediately.

On extensile dorsal and volar releases, copious frank purulence was encountered (Figure 4). Intraoperatively, a standard serpentine volar incision was extended proximally across the antecubital fossa in a “lazy S” fashion proximally. The mobile wad and proximal extensor mass were free of abscess. Proximally, the anatomy between the median nerve, brachial artery, and biceps tendon was dissected, and the abscess was found to track deep to the flexor pronator mass and distally along the radial and deep aspects of the flexor pollicis longus. Periosteum about the radius was denuded, and the bare radius was exposed. The abscess perforated through the interosseous membrane dorsally, where on dorsal incision, the abscess was found to track along the fourth dorsal compartment. Pathological gross intraoperative findings included muscular changes including edema, displacement, and necrosis localized at the pronator quadratus and erosion of the interosseous membrane at the distal forearm. The extent of bony and muscular necrosis encountered at the distal radius, along with localized periosteal stripping, suggested a nidus at the distal radius. The forearm was débrided thoroughly and copiously irrigated, and the surgical incisions were left open with wet-to-dry oxychlorosene sodium–soaked dressings for planned serial débridement. He was admitted to the general pediatrics service with orthopaedics and pediatric infectious disease consulting. He was started on empiric vancomycin and piperacillin-tazobactam pending surgical culture speciation and sensitivities. He underwent repeat irrigation and débridement on hospital days 2, 4, and 7, with split thickness skin grafting done during final débridement. Intraoperative cultures grew methicillin-resistant *Staphylococcus aureus* (MRSA) susceptible to trimethoprim-sulfamethoxazole and rifampin. Daily CRPs were monitored and ultimately corrected to 2.3 mg/dL before discharge on hospital day 10. He was discharged on an oral antibiotic regimen consisting of double-strength trimethoprim-sulfamethoxazole and rifampin.

**Figure 5:**
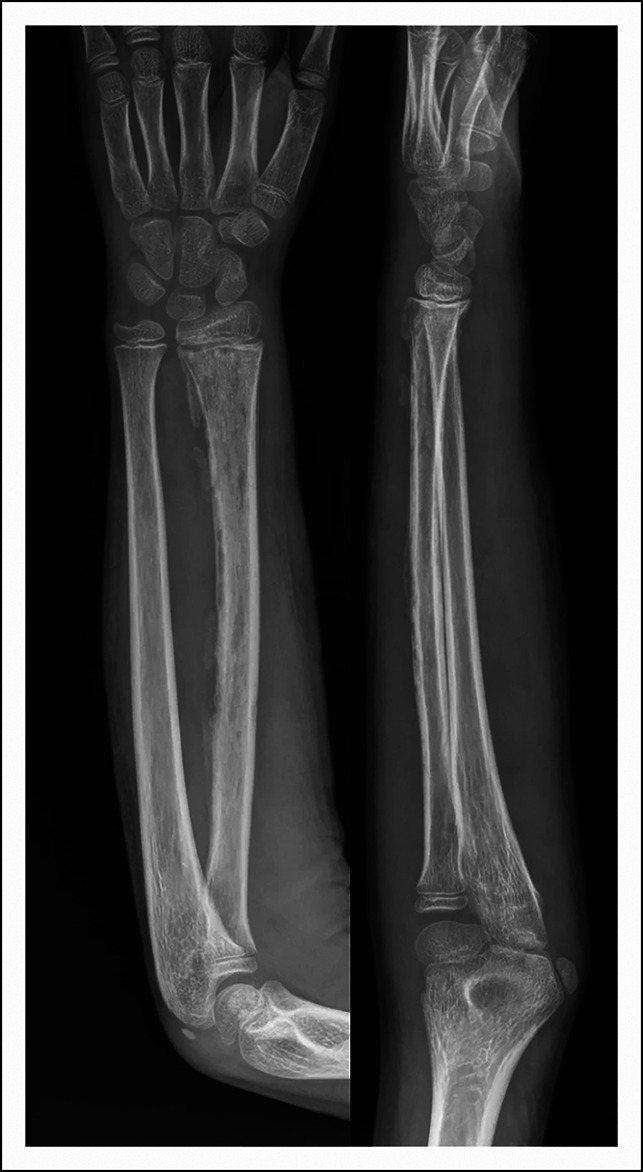
AP and lateral radiographs of the left forearm with regional osteopenia, periosteal reaction, and radial cortical erosion suggestive of osteomyelitis.

The patient presented to the orthopaedics clinic 1 week after discharge and had recovered full AIN function and partial PIN function. Thirteen days after discharge, the patient represented to the pediatric emergency department with 2 days of fever, maximum 103.5 F, persisting despite NSAID administration. He maintained compliance with oral trimethoprim-sulfamethoxazole and rifampin and denied any wound complications. Home care nursing had done dressing changes 3 times per week. Physical examination demonstrated well-healing skin graft and surgical incisions without drainage or dehiscence. He had regained full AIN, PIN, and ulnar nerve function. At the time of representation, the white blood cell count was 7.8 k/mcL, erythrocyte sedimentation rate was 120 mm/hr, and CRP was 14.8 mg/dL. Radiographs of the forearm demonstrated persistent OM (Figure 5). Contrast-enhanced MRI demonstrated OM of the radius and ulna without abscess. No surgical intervention was recommended; however, he was admitted to the general pediatrics service for treatment of ongoing OM. He was started on intravenous vancomycin and continued oral rifampin. Infectious disease trended CRP daily on intravenous antibiotics. After CRP improvement to 3.3 (hospital day 3), he was transitioned to oral linezolid and rifampin and was discharged. Per telemedicine discussion with the father 8 months postoperatively, the patient had regained full function of his elbow, wrist, and hand; was participating in recreational sports; and had no residual complaints.

## Discussion

### Acute Hematogenous Osteomyelitis

Acute hematogenous OM and septic arthritis occur in approximately 1:10,000 children younger than 12 years old per year.^9^ Common pathogens include *S aureus* (25% to 60% of cases), group A Streptococcus, group B Streptococcus, and *Haemophilus influenzae* group B (historically and in unvaccinated children). Community-acquired MRSA infections have become a leading cause of pediatric acute hematogenous OM; risk factors include African American race, Medicaid enrollment, and previous systemic infection.^10^ MRSA osteoarticular infections are more severe than methicillin-susceptible *S aureus*, require longer stays in the hospital, are more likely to have abscess requiring surgical débridement, and are more likely to have complications such as septic thrombophlebitis and septic pulmonary emboli.^11^ Pain is the hallmark presenting symptom in acute hematogenous OM. Extremity pain with an inability to move or bear weight on the limb should suggest OM. Similar to this case, younger patients often present with poorly localized pain, whereas older children better localize pain.^12^ In acute hematogenous OM, classic signs of inflammation including swelling, erythema, and warmth are often absent unless the infection eroded the cortex entering the subperiosteal space, as was seen in this case.^13^ This patient's clinical examination correlates to intraoperative findings including diffuse subperiosteal abscess with erosion through the interosseous membrane. We hypothesize that this patient sustained a nondisplaced Salter-Harris type I fracture during his initial fall, with seeding of fracture hematoma during his transient fever on postinjury day 2. The subperiosteal abscess developed and eroded through soft tissues including the interosseous membrane, accumulating such volume that intracompartmental pressures exceeded perfusion pressure causing acute CS.

### Development of Compartment Syndrome

CS, the development of pressure in the compartments such that perfusion pressure is exceeded and impairs circulation, most commonly occurs secondary to trauma. Rarely, CS occurs after burns and intracompartmental bleeding—especially in anticoagulated patients, embolic events, and infection.^14^ The forearm is the most common site for upper extremity CS, most often secondary to supracondylar humerus fractures and both bone forearm fractures.^3,15^ Acute CS has been reported in the setting of acute hematogenous OM, and a subacute history of trauma is often present.^16^ Acute CS has been reported as a rare consequence of chronic OM after fracture.^17^

Despite its rarity, consequences of delayed diagnosis and management of CS after acute hematogenous OM remain: neurologic compromise, Volkmann ischemic contractures, infection of devitalized tissues, loss of function, and possible loss of limb.^18^ Given the severity of missed or delayed diagnosis, it is critical to consider CS despite atraumatic presentations.

In this case, clinical examination demonstrating tense compartments, evolving neurologic deficit, and flexion contracture at the elbow were all consistent with CS; however, examination and diagnosis of CS in children can be difficult. The classic “five P's of CS”—pain, paresthesias, paralysis, pallor, and pulselessness— are difficult to assess in children. In pediatric patients, increasing pain, anxiety, or agitation and increasing analgesic requirements are often the best indicators of an evolving CS.^4^ Finally, compartment pressure measurements can be useful in diagnosing CS in very young patients, sedated patients, or obtunded pediatric patients; however, needle measurements are anxiety-provoking and painful, and risks must be weighed against potential diagnostic gain.

Once acute CS is diagnosed, emergent fasciotomies are indicated. In the forearm, carpal tunnel release is often concomitantly indicated. Fracture-related forearm CS commonly requires a single palmar incision to facilitate superficial and deep palmar compartment release, as well as carpal tunnel release. In this case, intercompartmental abscess eroded through the interosseous membrane into the dorsal compartment requiring additional dorsal release.

## Conclusion

CS resulting from hematogenous OM is rare; however, rapid recognition, diagnosis, and intervention are imperative given the potential consequences of delayed or missed diagnosis. At the time of initial orthopaedic evaluation, this patient demonstrated neurologic dysfunction evidenced by his complete AIN and incomplete PIN palsies. Diagnosing CS in a pediatric patient largely remains a clinical diagnosis; however, pediatric CS presentations often differ from adult presentations. Although this patient successfully recovered function postoperatively, delay in diagnosis or management may lead to irreversible neurologic deficit, permanent ischemic contractures, or even loss of limb, emphasizing the criticality in considering acute CS despite atraumatic presentations.
